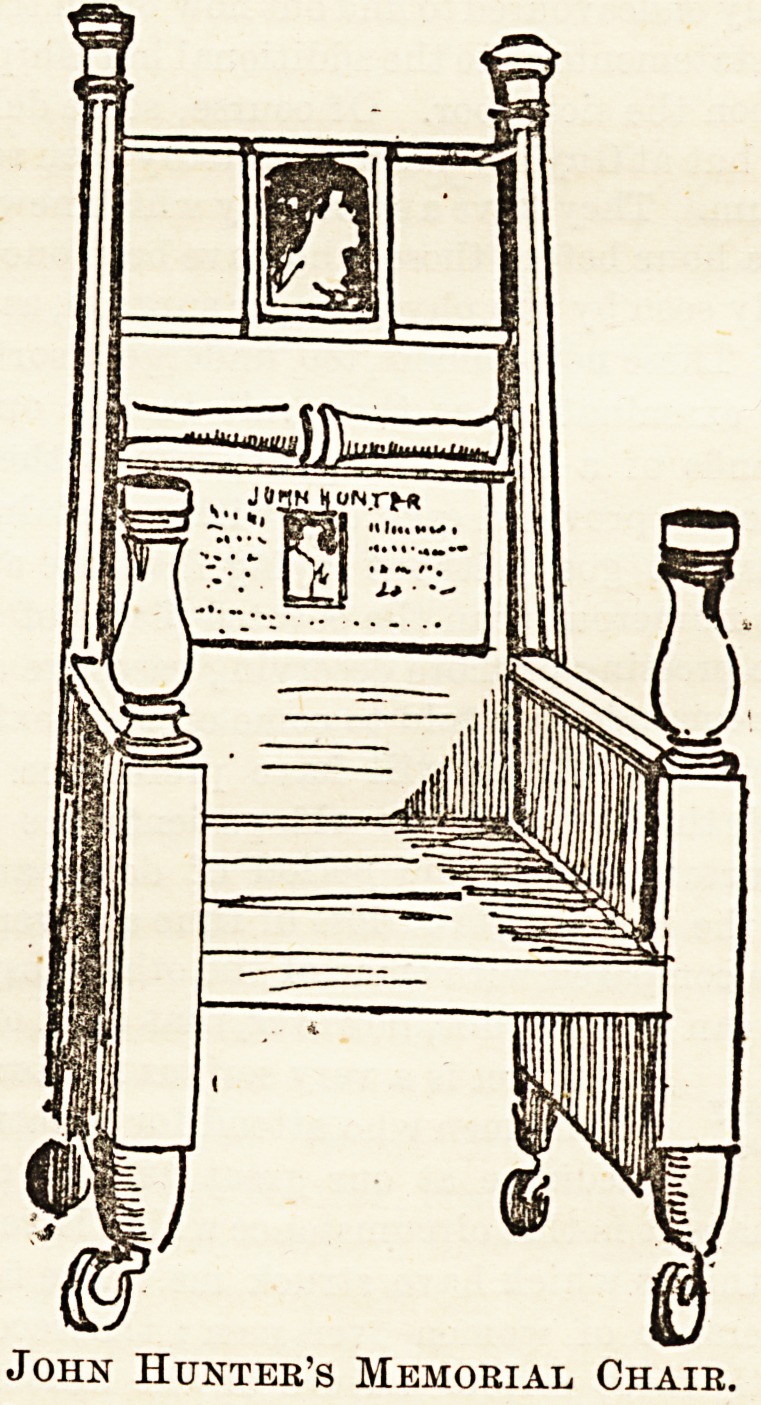# A Relic of John Hunter

**Published:** 1887-02-12

**Authors:** 


### Frank Buckland's Relic of John Hunter.

The Royal College of Surgeons, in Lincoln's Inn Fields, has recently been presented by Mrs. Buckland (the widow of the late Frank Buckland) with a most interesting relic of the great physiologist and surgeon, John Hunter. The relic, which is now in the form of a chair, was originally an imposing-looking bedstead, of the old-fashioned four-post order. It was on this bed that the distinguished founder of our system of modern surgery slept for many years when a resident of Earl's Court. Hunter, as most of our readers are doubtless aware, fell dead on the floor of St. George's Hospital, consequent on the excitement induced by a meeting he had just attended. His remains were brought from the hospital to Eail's Court, and placed, prior to removal for burial, o this old bed. The tragic event happened 17th Octo[illegible]r, 1793. The bed subsequently became the property of Sir Richard (then Professor) Owen, who, in turn, gave it to the late Mr. Buckland, a staunch admirer of John Hunter. "For several years," writes Mr. Henry Ffennell in a contemporary, "the cumbrous frame lay in the house in Albany Street which Mr. Buckland occupied for so many years, until he formed the idea of converting it into a chair, which could be conveniently moved about and shown to visitors. He immediately set to work, and, with the assistance of his secretary, Mr. Searle, plans were made, measurements taken, and the limbs of the huge frame cut up. When, however, the amateur cabinet-makers came to set up the frame of the chair, they were completely beaten, and they had to call in the assistance of a professional, who in due time finished the work to the satisfaction of all parties concerned." Such, then, is the history of the old relic, of which, by the courtesy of the proprietors of *The Pall Mall Gazette*, we are able to furnish our readers with a sketch. Mrs. Buckland, who since her husband's death has carefully treasured the chair, recently made an offer of it to the College of Surgeons, and the Council have accordingly placed it in the Museum, in fitting companionship with so much else which is sacred to the name of Hunter.

It was mainly through the efforts of the late Mr. Buckland that the remains of John Hunter were removed from the vaults of St. Martin's-in-the-Fields to Westminster Abbey. "A few months before Mr. Buckland's death," writes Mr. Ffennell, "I spent one day a very pleasant couple of hours with him in Westminster Abbey, when he told me several amusing stories connected with his own life at Westminster, when he

John Hunter's Memorial Chair.

resided there during the time his father was Dean. Coming to where the remains of John Hunter repose, he said to me, 'I feel prouder of being the means of laying John Hunter's remains here than of anything I ever did in my life.'"

The upper inlet into the panel at the back of the chair is a photograph of a statue of Hunter in the Museum of the College, and the lower one is a bust of Hunter. This is accompanied by the following inscription: "This chair is made from the bedstead of John Hunter: born, February 14, 1728; died, October 17, 1793; buried at St. Martin's-in-the-Fields, Charing Cross; reinterred, Westminster Abbey, 1850. Was first used by Dr. Wadham, president at the annual dinner, St. George's Hospital, October 1, 1879. The bedstead was given to me (as a birthday present) by Professor Owen, F.R.S., who writes: 'This is a genuine relic of John Hunter. Mr. W. Clift bought it at the sale of furniture in Leicester Square. It is the frame of the bedstead on which John Hunter lay when brought from St. George's Hospital.—Frank Buckland, M.A., M.R.C.S., Inspector of Fisheries, House Surgeon, 1851.'"

## Figures and Tables

**Figure f1:**